# Cutaneous Adverse Events of Tyrosine Kinase Inhibitors in Endocrine Tumors: Clinical Features, Mechanisms, and Management Strategies

**DOI:** 10.3390/biomedicines13123044

**Published:** 2025-12-11

**Authors:** Marta Marino, Francois Rosset, Alice Nervo, Alessandro Piovesan, Valentina Pala, Elisa Vaccaro, Luca Mastorino, Aldo E. Calogero, Emanuela Arvat

**Affiliations:** 1 Department of Clinical and Experimental Medicine, University of Catania, 95123 Catania, Italy; aldo.calogero@unict.it; 2 Oncological Endocrinology Unit, AOU Città della Salute e della Scienza, University of Turin, 10126 Turin, Italy; alice.nervo@gmail.com (A.N.); apiovesan@cittadellasalute.to.it (A.P.); elisa.vaccaro@unito.it (E.V.); emanuela.arvat@unito.it (E.A.); 3 Dermatology Department, San Lazzaro Hospital, University of Turin, 10126 Turin, Italy; francois.rosset95@gmail.com (F.R.); v.pala@unito.it (V.P.); luca.mastorino@unito.it (L.M.)

**Keywords:** protein kinase inhibitors, thyroid neoplasms drug therapy, neuroendocrine tumors drug therapy, skin diseases drug therapy, drug-related side effects and adverse reactions, quality of life

## Abstract

**Background:** Tyrosine kinase inhibitors (TKIs) are crucial to treating endocrine-related malignancies, including advanced thyroid cancers and neuroendocrine tumors, but their benefit is tempered by cutaneous adverse events (CAEs) that impair adherence and quality of life. **Objective:** To summarize the dermatologic toxicities of TKIs used in endocrine oncology and provide practical, multidisciplinary guidance for prevention and management. **Methods:** Narrative synthesis of clinical trial reports, post-marketing studies, and specialty guidelines pertinent to lenvatinib, vandetanib, cabozantinib, and other commonly used TKIs, integrating dermatologic and endocrine perspectives on mechanisms and care pathways. **Results:** VEGFR-targeted TKIs frequently cause hand–foot skin reaction, xerosis, fissuring, paronychia, and impaired wound healing; multikinase inhibition also produces alopecia, pigmentary changes, and mucositis. Epidermal growth factor receptor (EGFR) and rearranged during transfection (RET) inhibition with vandetanib is associated with acneiform eruption, photosensitivity, and nail fragility. Pathogenesis reflects on-target inhibition of VEGF/EGFR signaling leading to keratinocyte dysfunction, vascular fragility, and altered eccrine mechanics. Early risk stratification, patient education, and bundle-based prophylaxis (emollients, keratolytics, urea-based creams, sun protection) reduce incidence and severity. Grade-based algorithms combining topical corticosteroids/antibiotics, dose interruptions or reductions, and short systemic courses (e.g., doxycycline, antihistamines) enable symptom control while maintaining anticancer intensity. Close coordination around procedures minimizes wound-healing complications. **Conclusions:** Dermatologic toxicities are predictable, mechanism-linked, and manageable with proactive, multidisciplinary care. Standardized prevention and treatment pathways tailored to specific TKIs—particularly lenvatinib, vandetanib, and cabozantinib—can preserve dose intensity, optimize quality of life, and sustain antineoplastic efficacy.

## 1. Introduction and Scope of the Review

Tyrosine kinase inhibitors (TKIs) have become crucial in the treatment of various endocrine malignancies, particularly differentiated and medullary thyroid carcinomas and gastro-entero-pancreatic neuroendocrine tumors [1,2]. Their therapeutic efficacy, however, is frequently associated by cutaneous adverse events (CAEs), which range from mild, self-limiting symptoms to severe toxicities that may compromise treatment adherence or require dose modification, thus potentially reducing efficacy of anti-neoplastic treatment [3,4].

Among the most observed reactions are hand–foot skin reaction (HFSR), xerosis with eczema-like changes, pruritus, nail and hair abnormalities, and pigmentary disturbances. These manifestations are not only drug- and class-dependent—frequent, for instance, with vascular endothelial growth factor receptors (VEGF-R) targeting TKIs—but also influenced by patient-specific factors and cumulative exposure over time [5]. In endocrine oncology, where therapies are often administered chronically and disease control may rely on long-term treatment continuity, CAEs can significantly affect both quality of life and oncologic outcomes [6].

Despite increasing recognition of these toxicities in general oncology literature, data specifically focused on endocrine tumors remain limited and are often extrapolated from broader populations [7]. This narrative review aims to fill that gap by summarizing the clinical spectrum of TKI-induced CAEs in endocrine tumors, exploring their underlying mechanisms, and proposing practical, mechanism-informed management strategies to support early recognition and proactive care.

## 2. Materials and Methods

### 2.1. Review Design

We conducted a narrative, multidisciplinary review integrating endocrinology and dermatology perspectives to summarize cutaneous adverse events (CAEs) associated with tyrosine kinase inhibitors (TKIs) used in endocrine tumors. The review emphasizes clinical manifestations, pathophysiology, and prevention/management strategies relevant to routine care. No protocol was preregistered.

### 2.2. Data Sources and Search Strategy

We searched PubMed/MEDLINE, Scopus, and Web of Science from database inception to 1 September 2025. Searches combined controlled vocabulary and keywords related to TKIs and endocrine oncology with dermatologic outcomes. A representative PubMed string was: (“tyrosine kinase inhibitor” OR TKI OR lenvatinib OR vandetanib OR cabozantinib OR sorafenib OR sunitinib OR selpercatinib OR pralsetinib OR larotrectinib OR entrectinib OR dabrafenib OR trametinib) AND (thyroid OR medullary OR “radioiodine-refractory” OR endocrine OR neuroendocrine OR NET OR “gastroenteropancreatic”) AND (cutaneous OR dermatologic OR skin OR “hand–foot” OR HFSR OR paronychia OR xerosis OR pruritus OR photosensitivity OR mucositis). Searches were supplemented by manual screening of reference lists and relevant guidelines or position statements.

### 2.3. Eligibility Criteria

Population: adults with endocrine-related malignancies (e.g., differentiated or medullary thyroid carcinoma; gastroenteropancreatic neuroendocrine tumors) receiving TKIs. Interventions/agents: multikinase inhibitors (e.g., lenvatinib, sorafenib, cabozantinib, sunitinib) and selective inhibitors (e.g., RET, neurotrophic tyrosine receptor kinase (NTRK), BRAF/MEK (mitogen-activated protein kinase pathway)) commonly used in endocrine oncology. Outcomes: incidence, timing, and severity of CAEs; mechanistic insights; prophylaxis and management, including dose modifications. Designs: randomized trials, prospective or retrospective cohorts, registries, meta-analyses, and case series with n ≥ 5. For rare, severe events (e.g., Stevens–Johnson syndrome/toxic epidermal necrolysis (SJS/TEN), drug reaction with eosinophilia and systemic symptoms (DRESS)), informative single-case reports were considered. Exclusions: non-clinical studies without translational relevance; non-English full texts when adequate translation was unavailable; abstracts without extractable data. High-quality data from non-endocrine oncology were included when class effects or mechanisms were directly applicable.

### 2.4. Study Selection

Two reviewers independently screened titles and abstracts and assessed full texts against eligibility criteria. Disagreements were resolved by consensus with a third reviewer. Reasons for exclusion were recorded at full-text stage.

### 2.5. Data Extraction

Using a standardized template, we collected tumor type, TKI and dosing, study design and size, CAE definitions, grading system, incidence and severity, time to onset, management (topical and systemic therapies, prophylaxis bundles), dose interruptions or reductions, outcomes (symptom control, adherence, oncologic endpoints), and follow-up duration.

### 2.6. Definitions and Grading

Dermatologic events and severity were described using the Common Terminology Criteria for Adverse Events (CTCAE) version 5.0, with harmonization of synonyms (e.g., hand–foot skin reaction vs. palmar–plantar erythrodysesthesia) to maintain consistency across sources.

### 2.7. Synthesis Approach

Findings were synthesized qualitatively and organized by predominant clinical phenotype (e.g., HFSR, xerosis/eczema, papulopustular eruptions, pruritus, nail disorders, pigmentary changes, photosensitivity, mucositis) and by putative mechanism (angiogenesis blockade; MAPK/EGFR/RET pathway effects; neuroimmune dysregulation). When data allowed, we prioritized endocrine-specific studies and corroborated management recommendations across trial reports, post-marketing data, and specialty guidelines.

### 2.8. Ethics

No human subjects were directly involved; ethical approval and informed consent were not applicable.

## 3. Background

The introduction of TKIs has significantly changed the therapeutic landscape for several endocrine malignancies [5]. Advanced differentiated thyroid carcinoma (DTC), medullary thyroid carcinoma (MTC), and well-differentiated gastro-entero-pancreatic neuroendocrine tumours (GEP-NETs) represent key indications where TKIs have demonstrated clinical benefit [8,9]. TKIs are cytostatic agents, thus useful to achieve stabilization of disease, not remission, and they are reserved for progressive, symptomatic, or organ-threatening diseases [10]. In these settings, disease progression often occurs indolently, making long-term disease control and treatment tolerability essential priorities [11]. Due to their multiple targets among different tissues, systemic adverse event profiles differ, and dose adjustments are often required to manage toxicity [12].

The TKIs used in endocrine tumors can be broadly divided into two categories: multi-target agents, which commonly inhibit VEGF-R, platelet-derived growth factor receptors (PDGF-R), fibroblast growth factor receptor (FGF-R), and c-KIT, and selective inhibitors that target specific oncogenic drivers such as RET (Rearranged during Transfection), Neurotrophic Tyrosine Receptor Kinase (NTRK), or BRAF. Lenvatinib and sorafenib are multikinase inhibitors approved for the treatment of radioiodine-refractory DTC, including papillary and follicular subtypes [13]. Lenvatinib is generally considered the preferred first-line agent due to superior progression-free survival, but both agents are widely used [14]. Cabozantinib is approved for DTC as a second-line agent after progression on other TKIs, and for advanced MTC [15]. For anaplastic thyroid cancer with BRAF V600E mutation, the combination of dabrafenib plus trametinib (BRAF/MEK inhibitors) is FDA-approved [16]. Selective RET inhibitors as selpercatinib and pralsetinib have shown high response rates and improved tolerability in RET-mutated thyroid cancers, while larotrectinib and entrectinib target NTRK fusions across tumor types, including rare thyroid cancer subtypes [17]. For advanced GEP-NETs, sunitinib is currently approved in pancreatic NETs [8]. For advanced GEP-NETs, most used TKIs are sunitinib and cabozantinib, with additional investigational agents including lenvatinib, surufatinib, axitinib, and pazopanib [18]. Each of these agents carries a distinct but overlapping dermatologic toxicity profile. For instance, VEGFR inhibition is strongly associated with hand-foot syndrome (HFS), xerosis, and nail disorders, while selective RET or NTRK inhibitors may cause rash, pruritus, or pigmentary changes—often with a milder overall profile but longer cumulative exposure [19]. Understanding these patterns is essential for anticipating adverse events, tailoring preventive strategies, and guiding appropriate dermatologic interventions.

The incidence of CAEs associated with TKIs in endocrine neoplasms is high, with ≥90% of patients experiencing at least one adverse event of any grade during therapy [3]. HFSR occurs in up to 37% of patients treated with sorafenib, 54% with cabozantinib, and is also common with sunitinib and lenvatinib; rash is seen in up to 41% with vandetanib; pruritus occurs in 14% with sorafenib; xerosis and pigmentary changes are reported in 20–44% of patients on cabozantinib [20,21]. Dose reduction due to cutaneous toxicity may be required in approximately 30% of patients, and treatment discontinuation occurs in about 13% [20]. From a dermatologic standpoint, anti-VEGFR multikinase inhibitors are mainly associated with hand–foot skin reaction (HFSR), xerosis/fissuring and onychopathies, whereas selective RET and NTRK inhibitors tend to induce hypersensitivity-type rash, pruritus and pigmentary alterations, generally of milder grade but often prolonged over time [20,21]. The impact of dose reduction or discontinuation on antineoplastic efficacy and overall survival still has a nuanced perspective. While temporary dose reductions or interruptions can ameliorate symptoms and allow continuation of therapy, permanent discontinuation may compromise disease control and survival. However, evidence suggests that dose reductions do not necessarily abrogate efficacy if managed appropriately, as maintaining patients on therapy—even at reduced doses—can preserve clinical benefit [4,12]. Early recognition and management of cutaneous toxicities are essential to optimize outcomes and minimize the risk of premature discontinuation [3,4,12].

## 4. Major Manifestations

CAEs associated with TKIs in endocrine tumors encompass a broad array of dermatologic toxicities, varying in onset, severity, and pathogenesis. While most are low to moderate in grade, they may become dose-limiting in the absence of timely recognition and management. The clinical presentation of CAEs is influenced by the pharmacologic target profile of each TKI, patient-specific factors, and environmental or mechanical triggers. Below are the most relevant and characteristic manifestations.

### 4.1. Hand–Foot Skin Reaction (HFSR)

**Figure 1:**
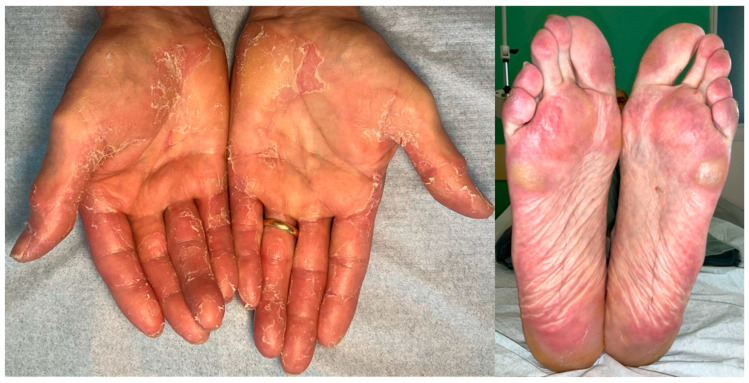
Hand-Foot Skin Reaction in a patient treated with Lenvatinib. Note the typical involvement of pressure point on palms and soles.

HFSR is a hallmark dermatologic toxicity associated with VEGFR-targeting TKIs such as sorafenib, lenvatinib, cabozantinib, and sunitinib [22]. It typically presents within 2 to 6 weeks of treatment initiation and affects pressure-bearing areas of the palms and soles. Clinically (Figure 1), it manifests as sharply demarcated painful erythema, often accompanied by hyperkeratosis, blistering, desquamation, and fissures localized to areas subjected to repetitive friction—such as the heels, metatarsal heads, and fingertips [23].

Unlike the diffuse palmar-plantar erythrodysesthesia (PPE) seen with classic chemotherapeutic agents like capecitabine, HFSR lesions are more localized and often asymmetric. Histologically, affected skin shows parakeratosis, acanthosis, keratinocyte vacuolization, and perivascular lymphocytic infiltrates. The pathogenesis involves compromised dermal microcirculation secondary to VEGFR and PDGFR inhibition, resulting in impaired vascular repair and localized ischemia exacerbated by mechanical stress [24].

Grading of HFSR follows the CTCAE criteria: grade 1 involves minimal skin changes without pain, grade 2 includes painful lesions with functional impairment, and grade 3 indicates severe pain or ulceration that significantly limits daily activities [25]. Although often perceived as a nuisance toxicity, HFSR has been associated with improved oncologic response in some studies, potentially serving as a pharmacodynamic biomarker [26]. Management requires a preventive and therapeutic approach. Prophylactic strategies include the use of urea- or salicylic acid–based keratolytics, emollients, protective gloves or footwear, and mechanical offloading of pressure zones [20]. Once lesions appear, topical high-potency corticosteroids, analgesics, and selective debridement may be necessary. In severe cases, temporary interruption of the TKI and subsequent dose adjustment may be required to maintain adherence without compromising treatment efficacy [4].

### 4.2. Xerosis, Eczematous Dermatitis, and Fissuring

Xerosis is one of the most frequently encountered but often underappreciated dermatologic toxicities of TKIs, particularly those that interfere with epidermal signaling such as EGFR, RET, and MAPK-related pathways [21,27]. Clinically, xerosis presents as dry, rough, scaly skin, with a predilection for the extremities and trunk. In more advanced cases, fissuring, painful cracks, and eczematous changes can develop, often complicated by secondary infections [28]. The pathophysiology involves disruption of epidermal homeostasis due to impaired keratinocyte proliferation and differentiation, reduced sebaceous activity, and altered lipid synthesis. This results in increased trans-epidermal water loss and barrier dysfunction, predisposing the skin to irritation and inflammation [27]. In cold or low-humidity climates, or in patients with pre-existing atopic diathesis, the condition may be further exacerbated. Histologically, xerotic and eczematous areas may show orthokeratosis, epidermal thinning or spongiosis, and superficial inflammatory infiltrates. Management relies on aggressive skin hydration with lipid-rich emollients, urea-based moisturizers, and occlusive formulations. In cases of eczematous transformation, medium- to high-potency topical corticosteroids or calcineurin inhibitors may be employed. Regular skin care counseling and climate adaptation strategies (e.g., humidifiers) are essential for long-term control [29].

### 4.3. Maculopapular and Urticarial Eruptions

Maculopapular eruptions are commonly reported across multiple TKI classes and tend to appear during the first month of therapy [4]. These lesions typically consist of erythematous macules and papules, symmetrically distributed on the trunk and limbs, often accompanied by mild pruritus. While most cases are self-limiting, extensive eruptions may cause significant discomfort and anxiety for the patient [3]. The underlying mechanism is thought to involve inflammatory activation in the epidermis and dermis due to altered signaling in keratinocytes, combined with subclinical barrier impairment. In contrast, urticarial or angioedema-like reactions are less frequent and may mimic immune-mediated hypersensitivity, especially if associated with systemic symptoms such as fever or eosinophilia. However, most TKI-induced eruptions are non-IgE–mediated and histologically display a superficial perivascular lymphocytic infiltrate, with or without eosinophils [30]. Treatment typically includes topical corticosteroids and nonsedating antihistamines. Systemic corticosteroids may be indicated for grade ≥2 reactions or when discomfort interferes with therapy. Skin biopsy is rarely required but may aid diagnosis in atypical or persistent presentations [12].

### 4.4. Pruritus

Pruritus is a frequent and often underestimated symptom in patients receiving TKIs [21]. It may occur with or without visible skin lesions and can range from mild to debilitating. Generalized or localized pruritus may begin early in therapy or arise later as a chronic manifestation, and its intensity does not always correlate with the extent of visible skin involvement [29].

The etiology is multifactorial. Cytokine dysregulation—particularly increased levels of IL-31, IL-6, and substance P—has been implicated in pruritus pathogenesis, along with neurogenic inflammation and damage to peripheral nerve endings. Epidermal barrier impairment may further facilitate pruritogen penetration and sensory nerve stimulation. Neuropathic pruritus has also been proposed, especially in cases unresponsive to antihistamines [31].

Effective management begins with optimizing skin hydration and treating underlying xerosis. First-generation antihistamines may be helpful for nocturnal symptoms, though their overall efficacy is limited. In resistant cases, topical menthol, pramoxine, or even gabapentinoids may be considered [32]. It is important to rule out systemic causes such as renal or hepatic dysfunction, particularly with TKIs that undergo hepatic metabolism or affect VEGF pathways [3].

### 4.5. Nail Disorders and Paronychia

Nail toxicities are well-recognized in the context of multikinase inhibitors and may include onycholysis, brittle nails, splinter hemorrhages, and most characteristically, paronychia [33]. Paronychia typically affects the lateral nail folds of the fingers or toes, often presenting after several weeks of treatment. It may evolve into painful swelling, erythema, and overgrowth of friable granulation tissue, sometimes complicated by bacterial or fungal infection [34]. The pathophysiology of TKI-induced paronychia involves disordered keratinocyte proliferation, impaired repair of periungual tissues, and repeated trauma from daily activities. VEGFR and EGFR inhibition may contribute by altering epithelial turnover and local immunity. Risk increases with prolonged therapy and poor nail hygiene [34].

Management includes mechanical offloading, antiseptic soaks (e.g., diluted acetic acid or chlorhexidine), topical corticosteroids, and silver nitrate application for granulation tissue. Silicone toe caps or finger sleeves may reduce trauma [35]. In severe or infected cases, systemic antibiotics or temporary treatment interruption may be necessary. Chronic paronychia may persist for months even after TKI discontinuation, requiring long-term dermatologic support [36].

## 5. Rare but Severe Events

Although most CAEs associated with TKIs are mild to moderate in severity, potentially life-threatening reactions can occur. These events often require prompt recognition, discontinuation of the offending agent, and multidisciplinary management, including dermatology, oncology, and in some cases internal medicine or intensive care.

### 5.1. Stevens–Johnson Syndrome and Toxic Epidermal Necrolysis

Most critical severe toxicities are Stevens–Johnson Syndrome (SJS) and Toxic Epidermal Necrolysis (TEN). These acute mucocutaneous syndromes are characterized by rapidly progressing epidermal necrosis, detachment, and mucosal involvement, often beginning with flu-like symptoms, fever, and painful erythematous macules or purpuric lesions [37]. Involvement of >30% of the body surface area defines TEN, while SJS affects less than 10% of the surface area [19]. Although extremely rare, cases have been reported with various TKIs, including sorafenib and vandetanib, sometimes in combination with other medications such as antibiotics or antiepileptics, which can increase the risk of immunologic skin reactions [38].

Histologically, these reactions show full-thickness keratinocyte apoptosis, subepidermal blister formation, and sparse lymphocytic infiltrates [37]. The suspected mechanism is a delayed-type hypersensitivity reaction mediated by cytotoxic T lymphocytes and natural killer cells. The presence of skin pain, mucosal erosions, and systemic symptoms should trigger immediate discontinuation of the TKI and referral to a specialized inpatient setting, preferably a burns or dermatology unit [39]. Management involves supportive care, fluid and electrolyte balance, wound care, and systemic immunomodulators such as corticosteroids, intravenous immunoglobulins, or cyclosporine, depending on institutional protocols and severity [19].

### 5.2. Drug Reaction with Eosinophilia

Drug Reaction with Eosinophilia and Systemic Symptoms (DRESS) is a rare but significant entity, also known as drug-induced hypersensitivity syndrome (DIHS). DRESS typically occurs 2 to 8 weeks after drug exposure and is characterized by a morbilliform eruption, facial edema, fever, eosinophilia, lymphadenopathy, and internal organ involvement—commonly the liver, kidneys, or lungs [40]. While most frequently associated with anticonvulsants and sulfonamides, DRESS has occasionally been reported with TKIs, particularly when used in combination with immunomodulatory drugs [41].

The pathophysiology involves a delayed immune reaction, often associated with viral reactivation (e.g., HHV-6), genetic predisposition (e.g., HLA alleles), and immune dysregulation. Diagnosis is clinical and supported by laboratory findings such as eosinophilia, atypical lymphocytes, and elevated liver enzymes [42]. The RegiSCAR scoring system can help standardize the diagnosis. Management includes immediate withdrawal of the culprit drug and systemic corticosteroids in moderate to severe cases. Reintroduction of the TKI is not recommended unless the risk–benefit balance strongly favors it and is done under close supervision [41].

### 5.3. Cutaneous Small-Vessel Vasculitis

Cutaneous small-vessel vasculitis, though rare, has been described in patients receiving VEGFR-targeting agents. It usually presents as palpable purpura on the lower limbs, occasionally with systemic involvement [43]. Biopsy typically shows leukocytoclastic vasculitis with perivascular neutrophilic infiltrates. In mild cases, withdrawal of the TKI alone may suffice, while in more severe or systemic cases, short courses of systemic corticosteroids may be required [44]. While these severe reactions are uncommon, their potential lethality demands high clinical suspicion and rapid action. Any sudden onset of widespread rash, blistering, mucosal erosions, or systemic symptoms in a patient on TKIs should be treated as a dermatologic emergency [45]. Early involvement of oncology-experienced dermatologist and, when indicated, inpatient care is essential to reduce morbidity and mortality. Importantly, re-challenge with the same TKI is contraindicated in most cases, and alternative agents with different chemical structures should be considered if continued oncologic therapy is necessary [28].

## 6. Main Pathogenetic Mechanisms of TKI-Related CAEs

**Figure 2:**
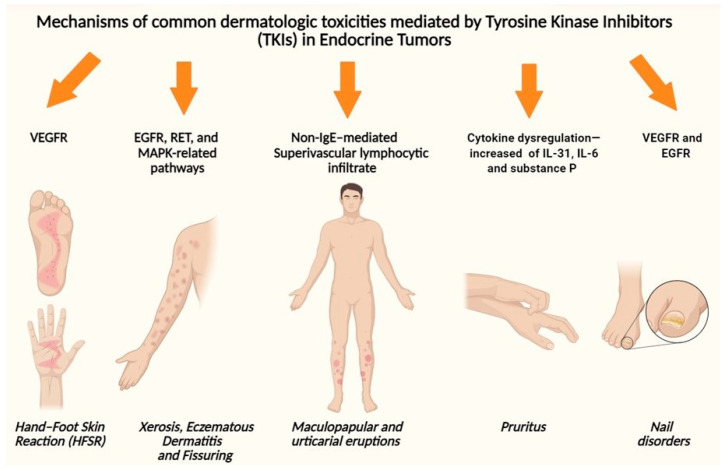
Proposed mechanism of most common CAE of TKI in endocrine tumors. Created in BioRender. Pala, V. (2025) https://BioRender.com/9b1z46t (accessed on 27 November 2025).

CAEs induced by TKIs reflect a complex interplay of pharmacodynamic effects on non-malignant tissues, involving skin, its annexes and microcirculation and its immune systems. Unlike cytotoxic agents damage proliferating cells indiscriminately, TKIs exert targeted effects on specific signaling cascades that, while driving tumor inhibition, also interfere with critical physiological pathways in healthy skin structures (Figure 2) [21].

A prominent and well-characterized mechanism involves inhibition of angiogenic signaling, particularly through blockade of VEGFRs, PDGFRs, and related tyrosine kinases. VEGFR is essential for maintaining dermal capillary networks and vascular permeability. Inhibition of this pathway reduces microvascular density, compromises skin perfusion, and impairs endothelial repair mechanisms [46]. Clinically, this manifests as HFSR, where the combination of localized mechanical stress and microcirculatory compromise in pressure-bearing areas leads to painful erythema, hyperkeratosis, and blistering. Reduced angiogenesis also affects wound healing, predisposing to fissures, ulcerations, and delayed resolution of traumatic or iatrogenic skin injury [20].

In addition to vascular effects, TKIs—particularly those targeting MAPK-related signaling such as RET, NTRK, and BRAF—disrupt epidermal homeostasis and adnexal function [21]. The MAPK/ERK pathway plays a pivotal role in keratinocyte proliferation, differentiation, and migration. Its inhibition can impair epidermal turnover and barrier integrity, leading to xerosis, scaling, and increased transepidermal water loss [35]. Furthermore, alterations in follicular signaling may cause hair texture changes, alopecia, or trichomegaly, while dysregulation of nail matrix and periungual epithelium contributes to onycholysis, paronychia, and periungual granulation tissue formation [28]. These effects are frequently observed with multikinase inhibitors like sorafenib, lenvatinib, or cabozantinib but also with newer selective agents depending on the molecular profile of the tumor (selpercatinib, larotrectinib, entrectinib) [47].

Another layer of pathogenesis involves inflammatory and neuroimmune dysregulation. Many CAEs, such as pruritus, eczematous eruptions, or urticarial lesions, may result from cytokine imbalances and local immune activation secondary to epidermal injury [41]. Pruritus often occurs in the absence of visible rash and may be mediated by IL-31, substance P, and other pruritogenic factors released by keratinocytes or sensory nerves following pathway inhibition [29]. The skin’s barrier dysfunction further facilitates antigen penetration and microbial colonization, potentially sustaining low-grade inflammation or superinfection. There is also growing interest in the role of cutaneous microbiome alterations, though data in the setting of TKIs remain preliminary [48]. Photosensitivity is another important, albeit underrecognized, toxicity mechanism—particularly with agents such as vandetanib or lenvatinib [49]. These reactions may be phototoxic, involving direct drug-induced generation of reactive oxygen species upon UV exposure, or photoallergic, involving delayed-type hypersensitivity [50]. Clinical presentations range from exaggerated sunburn reactions to eczematous or lichenoid lesions in photo-distributed areas [51].

Finally, host-related factors play a crucial modulating role in CAE development and severity. Age-related changes in skin structure, pre-existing dermatologic conditions (e.g., psoriasis, atopic dermatitis), vascular comorbidities (e.g., diabetes, peripheral artery disease), and concurrent medications (e.g., corticosteroids, anticoagulants) can all influence susceptibility to toxicity and the skin’s ability to recover from injury [23]. Genetic polymorphisms affecting drug metabolism or immune response pathways have also been proposed as potential contributors but remain to be validated in large cohorts [30].

## 7. Differential Diagnosis and Clinical Workflow

The diagnosis of CAEs in patients receiving TKIs requires a structured and pragmatic clinical approach. Many TKI-related dermatologic toxicities have overlapping features with other inflammatory, infectious, or immune-mediated skin conditions. Accurate identification is essential to initiate appropriate treatment, avoid unnecessary dose modification, and rule out severe or unrelated dermatoses [52]. The cornerstone of diagnostic evaluation is temporal correlation. Most TKI-induced CAEs appear within 2 to 8 weeks of treatment initiation, although some may manifest later during chronic therapy. Careful drug history—including recent dose escalations, concurrent medications, and prior dermatologic reactions—can help narrow the differential diagnosis. Importantly, polypharmacy is common in endocrine oncology, increasing the likelihood of interaction-based or synergistic toxicities.

The toxicity pattern varies in a predictable manner between multikinase VEGFR inhibitors and selective RET/NTRK agents, and should always be taken into account during clinical assessment [39,53].

Clinical examinations should be focused on morphology, distribution, and severity. Typical TKI-related rashes (e.g., HFSR, maculopapular eruptions, xerosis, paronychia) follow predictable patterns and topography. Conversely, atypical presentations—such as painful purpura, widespread bullae, or mucosal erosions—should raise suspicion for more severe entities like vasculitis, DRESS, or SJS/TEN [39,53]. Features such as systemic symptoms (fever, malaise), facial edema, lymphadenopathy, or internal organ involvement demand urgent evaluation [19].

Serial photography is a valuable tool, allowing objective monitoring of skin changes over time. This is particularly helpful in outpatient settings or during long-term therapy. When available, standardized tools (e.g., CTCAE grading scales) should be used to document lesion severity and guide treatment decisions.

Although many CAEs can be diagnosed clinically, ancillary investigations are sometimes necessary. Skin swabs or cultures may help identify secondary infections, especially in cases of fissures, ulcerations, or paronychia. Skin biopsy is rarely required for classic lesions but should be considered in cases with diagnostic uncertainty, rapid progression, or systemic involvement [54]. Histopathological findings can help distinguish TKI-related toxicities from primary dermatoses such as psoriasis, autoimmune bullous diseases, or vasculitis. For instance, leukocytoclastic vasculitis shows fibrinoid necrosis of vessel walls and neutrophilic infiltrates, while drug hypersensitivity reactions may present with interface dermatitis or apoptotic keratinocytes [13].

Differential diagnosis is particularly important in the setting of pre-existing skin conditions. Patients with atopic dermatitis, psoriasis, rosacea, or lichen planus may experience exacerbations under TKI therapy [55]. These flare-ups can mimic or overlap with drug-induced reactions and may require tailored dermatologic management rather than drug discontinuation. Furthermore, contact dermatitis, often due to topicals or protective devices used during therapy, may complicate the clinical picture and should be ruled out via patch testing or response to withdrawal of the suspected irritant.

Systemic conditions such as hepatic or renal dysfunction may also present with cutaneous signs, particularly pruritus or xerosis [56]. In such cases, laboratory assessment is essential to differentiate between drug-induced skin effects and paraneoplastic or metabolic causes. This is particularly relevant for patients on multitarget TKIs with known off-target hepatic or renal effects [20].

## 8. Practical Management by Manifestation and Dose-Modification Principles

Prophylactic application of emollients and keratolytics should be initiated before symptom onset, particularly in the case of lenvatinib and other high-risk TKIs. For prevention and daily moisturization, a 10–20% urea formulation is generally sufficient, whereas higher concentrations (e.g., 30–40%) may be used for short periods in the case of painful hyperkeratotic plaques.

During treatment if HSFR symptoms appear an integrated strategy that balances dermatologic symptom control with oncologic treatment goals. While most CAEs are manageable with topical or supportive therapies, some may require dose adjustments or temporary interruption of the TKI to prevent significant morbidity or discontinuation. A manifestation-specific, grading-based approach is recommended to ensure early intervention and maintain treatment adherence.

General principles include early patient education, baseline skin assessment, and the use of prophylactic measures—particularly in high-risk individuals or when initiating VEGFR-targeting agents. Clinical grading using CTCAE criteria provides a standardized framework for decision-making. Mild events (grade 1) are usually managed symptomatically, whereas moderate to severe events (grade ≥ 2) may necessitate escalation of therapy or to evaluate the need for dose interruption or reduction [25].

### 8.1. Management of HFSR

Prevention is key in HFSR. Patients should be advised to minimize friction and pressure on the hands and feet through cushioned footwear, soft socks, and avoidance of repetitive trauma. Prophylactic application of urea-based emollients (10–20%) and keratolytics can reduce hyperkeratosis and maintain skin pliability [57,58]. At the first suspicion of HFSR, mechanical offloading should be intensified, and high-potency topical corticosteroids (e.g., clobetasol propionate) applied twice daily to inflamed or painful areas. In grade 2 reactions, additional interventions such as lidocaine-containing creams, salicylic acid (6–10%) for keratotic plaques, and softening soaks may be helpful. Selective debridement of hyperkeratotic areas can reduce mechanical stress and pain [59]. For grade 3 HFSR with significant pain or ulceration, temporary interruption of the TKI is often necessary. Upon improvement to grade ≤1, the drug may be restarted at a reduced dose, following institutional or protocol-specific guidelines. In some cases, switching to an alternative TKI with a different toxicity profile may be considered if symptoms are refractory [54].

### 8.2. Management of Xerosis and Eczematous Dermatitis

Xerosis should be addressed pre-emptively with regular application of lipid-rich emollients containing ceramides, urea, or glycerine, applied at least twice daily. Avoidance of hot showers, soaps with high surfactant content, and physical irritants is recommended. If eczematous lesions or fissures develop, treatment with topical corticosteroids (class II–III) is indicated. In sensitive areas, calcineurin inhibitors such as tacrolimus may be preferred to minimize atrophy [60,61]. For painful fissures, barrier-repair ointments, hydrocolloid dressings, or silicone tapes may accelerate healing. Secondary infection should be suspected in the presence of exudate, erythema, or crusting and managed with appropriate topical or systemic antimicrobials [62].

### 8.3. Management of Maculopapular Eruptions

Most maculopapular eruptions respond to medium-potency topical corticosteroids (e.g., mometasone furoate) and oral nonsedating antihistamines. For grade 2 or higher reactions involving extensive areas or associated with pruritus, a short course of systemic corticosteroids (e.g., prednisone 0.5–1 mg/kg/day for 5–7 days) may be used. Lesions typically resolve with continued TKI treatment, though monitoring for progression is essential [63,64]. Skin biopsy is reserved for atypical presentations, suspected hypersensitivity, or lack of improvement. In severe reactions or in the presence of systemic symptoms, treatment interruption should be considered [65].

### 8.4. Management of Pruritus

Mild pruritus may be managed with regular skin hydration, colloidal oatmeal baths, and antihistamines. In cases with significant discomfort, menthol-containing creams or pramoxine may provide temporary relief. If symptoms persist despite these measures, second-line options include topical corticosteroids or gabapentinoids such as pregabalin or gabapentin, especially if a neuropathic component is suspected [31]. Persistent or generalized pruritus should prompt evaluation for hepatic or renal dysfunction. In cases where pruritus is debilitating and unresponsive, temporary TKI interruption may be warranted to confirm causality [66].

### 8.5. Management of Paronychia and Nail Disorders

Management of TKI-induced paronychia begins with mechanical protection and avoidance of trauma to the nail folds. Antiseptic soaks (e.g., diluted vinegar or chlorhexidine) can help control inflammation and bacterial overgrowth. Topical corticosteroids reduce periungual inflammation, and silver nitrate may be used to cauterize granulation tissue [67,68]. For more severe lesions, application of topical antibiotics (e.g., mupirocin) or antifungals may be necessary. In patients with exuberant or painful granulation tissue, treatment with pulsed dye laser or minor surgical debridement can be considered. If paronychia severely limits function or is associated with superinfection, temporary interruption or dose reduction in the TKI may be justified [20,69].

### 8.6. Management of Hair Disorders

Alopecia and hair texture changes are generally cosmetics but can significantly affect patient well-being. Counselling and expectation setting are crucial. In selected cases, topical minoxidil (2–5%) may promote regrowth or reduce hair shedding, although evidence in the context of TKIs is limited [70,71]. Trichomegaly or hypertrichosis typically does not require intervention unless causing ocular irritation, in which case trimming or referral to ophthalmology may be needed [72].

### 8.7. Management of Pigmentary Changes and Photosensitivity

Patients should be advised on rigorous photoprotection from the start of TKI therapy, including broad-spectrum sunscreen (SPF ≥ 50), physical barriers (clothing, hats), and behavior modification to avoid peak UV exposure. Hyperpigmentation or hypopigmentation is usually benign and may be reversible after drug discontinuation, though it can persist for months [35,49]. For patients with visible pigmentary changes, camouflage techniques or depigmenting agents (e.g., azelaic acid, hydroquinone under supervision) may be considered. Phototoxic or photoallergic reactions may require topical corticosteroids and temporary sun avoidance [56,72].

### 8.8. Management of Mucositis

Oral mucositis, cheilitis, or stomatitis can develop with TKIs, particularly multikinase agents. Patients should maintain strict oral hygiene with bland rinses (e.g., saline or bicarbonate), avoid irritants such as alcohol-based mouthwashes, and apply barrier agents (e.g., dexpanthenol gels) to affected areas. In symptomatic cases, topical anesthetics (e.g., lidocaine gel) and short courses of corticosteroids may be used [12]. If mucositis interferes with oral intake or worsens despite topical therapy, systemic analgesics or a temporary TKI hold may be required [73].

### 8.9. Management of Severe or Life-Threatening CAEs

For grade 3–4 CAEs, such as Stevens–Johnson syndrome, toxic epidermal necrolysis, or DRESS, the TKI must be permanently discontinued. Management should be coordinated with inpatient dermatology and internal medicine or critical care teams, depending on severity. Rechallenge is generally contraindicated, and alternative oncologic strategies should be discussed [19,74].

## 9. Conclusions and Key Messages

CAEs are common, multifactorial, and clinically impactful toxicities in patients receiving TKIs for endocrine tumors (Table 1). While most are low-grade and manageable, their chronicity and cumulative burden, particularly in long-term treatments such as those used for thyroid cancers and neuroendocrine tumors, demand a structured and proactive management approach. The spectrum of CAEs includes both class-specific toxicities, such as HFSR in VEGFR inhibitors, and broader cutaneous manifestations like xerosis, eczematous dermatitis, pruritus, nail disorders, and pigmentary changes. Rare but severe complications such as SJS/TEN and DRESS, though uncommon, must remain on the clinician’s radar due to their high morbidity. Early recognition, patient education, and consistent use of preventive measures—especially for high-risk toxicities such as HFSR and paronychia—are critical to avoid dose-limiting symptoms. A graded, mechanism-informed management strategy allows most patients to maintain full therapeutic intensity without compromising quality of life. Involving dermatology early, particularly in moderate-to-severe cases, ensures optimized outcomes and minimizes treatment discontinuation. Future studies specifically focused on endocrine tumor populations are needed to refine management algorithms, validate dermatologic biomarkers of response or toxicity, and develop targeted prevention strategies. Until then, a multidisciplinary, patient-centered approach remains the cornerstone of effective care.

## Figures and Tables

**Table 1:** Dermatologic adverse events with tyrosine kinase inhibitors (TKIs) in endocrine oncology: lesions, mechanisms, typical time to onset, and proposed management.

Lesion	Main Mechanisms	Typical Time to Onset	Proposed Management
Hand–Foot Skin Reaction (HFSR)	VEGFR/PDGFR inhibition → impaired dermal microcirculation; mechanical stress on pressure areas.	2–6 weeks after initiation.	Prophylaxis with emollients/urea 10–20% and keratolytics; mechanical off-loading; potent topical corticosteroids; salicylic acid for hyperkeratosis; topical anesthetics; selective debridement; for grade 3: temporary hold and restart at reduced dose.
Xerosis/eczematous dermatitis/fissures	Barrier dysfunction due to EGFR/RET/MAPK inhibition → reduced proliferation/lipid synthesis, ↑TEWL.	Early, often persistent/cumulative (first weeks–months).	Lipid-rich emollients (ceramides/urea/glycerin) ≥ 2/day; avoid irritants; class II–III topical corticosteroids or calcineurin inhibitors in sensitive areas; barrier dressings for fissures; topical/systemic antibiotics if superinfection.
Maculopapular eruption	Epidermal–dermal inflammation from altered signaling; typically, non–IgE mediated.	Usually within the first month.	Medium-potency topical corticosteroids + antihistamines; in grade ≥ 2 short courses of systemic corticosteroids; temporary interruption if severe/atypical; biopsy if diagnostic doubt.
Pruritus (with or without lesions)	Cytokines (e.g., IL-31), neurogenic inflammation, peripheral fiber damage; barrier dysfunction; possible neuropathic component.	Early or late; fluctuating course.	Intensive hydration; antihistamines (esp. at night); soothing topicals (menthol/pramoxine); consider gabapentinoids if refractory; rule out systemic causes (hepatic/renal).
Paronychia/nail disorders	Altered keratinocyte proliferation and periungual repair; microtrauma; VEGFR/EGFR involvement.	After several weeks of therapy.	Mechanical protection; antiseptic soaks (diluted vinegar/chlorhexidine); topical corticosteroids; silver nitrate for granulation; antibiotics/antimycotics if infection; laser/selective debridement; hold/dose reduction if severe.
Photosensitivity/pigmentary changes	Drug–UV interaction and epidermal dysfunction; reported with EGFR/RET inhibitors (e.g., vandetanib).	Variable; often with UV exposure during treatment.	Strict photoprotection (SPF ≥ 50, physical barriers, behavior); topical corticosteroids for phototoxic reactions; camouflage/depigmenting agents if needed; avoid UV until resolution.
Oral mucositis/cheilitis/stomatitis	Mucosal toxicity with multikinase inhibitors; altered mucosal barrier.	Variable; may impair oral intake.	Oral hygiene + bland rinses (saline/bicarbonate); barrier agents (dexpanthenol); topical anesthetics; short course of corticosteroids; systemic analgesics; hold TKI if severe.
Severe reactions (SJS/TEN; DRESS)	T cell–mediated hypersensitivity with keratinocyte apoptosis (SJS/TEN) or systemic immune response (DRESS).	DRESS 2–8 weeks; SJS/TEN often rapid with systemic symptoms.	Definitive TKI discontinuation; management in specialized setting; intensive support; immunomodulation (corticosteroids, IVIG, cyclosporine) as per protocols; re-challenge generally contraindicated.
Small-vessel cutaneous vasculitis	Leukocytoclastic vasculitis; reported with VEGFR inhibitors.	Variable; palpable purpura on lower limbs.	TKI discontinuation; systemic corticosteroids if extensive/systemic involvement; avoid re-challenge.

Abbreviations: TKI, tyrosine kinase inhibitor; VEGFR, vascular endothelial growth factor receptor; PDGFR, platelet-derived growth factor receptor; EGFR, epidermal growth factor receptor; RET, rearranged during transfection; HFSR, hand–foot skin reaction; TEWL, transepidermal water loss; CTCAE, Common Terminology Criteria for Adverse Events; SJS/TEN, Stevens–Johnson syndrome/toxic epidermal necrolysis; DRESS, drug reaction with eosinophilia and systemic symptoms.

## Data Availability

The data presented in this study were obtained from public domain resources such as PubMed, Scopus, and Web of Science.
